# Mapping Support-Seeking After Cancer Treatment: A Co-Designed Model of Triggers, Timing and Support Pathways in Young People with Lived Experience of Cancer

**DOI:** 10.3390/curroncol33070422

**Published:** 2026-07-15

**Authors:** Nicole Collaço, Anna Kennington, Natalie Greenberg, Tara Imber, Danae Warne, Samantha Sodergren

**Affiliations:** 1 Department of Behavioural Science and Health, University College London—UCL, London WC1E 7HB, UK; 2 School of Health Sciences, University of Southampton, Southampton SO17 1BJ, UK; s.c.sodergren@soton.ac.uk; 3 Patient Partner

**Keywords:** oncology, adolescent, young adult, support, help seeking, survivorship

## Abstract

Post cancer treatment, many young people often live with ongoing emotional, social, and physical difficulties, but support is not always accessed when it is needed. This study aimed to co-produce a conceptual model of support-seeking after cancer treatment with young people with lived experience of cancer, to better understand the triggers, timing, and pathways influencing engagement with support. This co-design work, informed by Bird et al.’s generative framework for co-production, built upon a prior study involving interviews and co-design workshops with young people and healthcare/allied health professionals, and informed a preliminary model of support-seeking. The current work involved two further co-production stages through an online survey and workshop to refine this model. Data were analysed using a thematic approach to support conceptual model development. Four interconnected themes shaped support-seeking: (1) readiness to engage: recognition, emotional readiness, and relational safety; (2) access and appraisal of support: visibility, fit, feasibility, and burden; (3) pathways to support: multi-modal, layered, and non-linear engagement; and (4) support trajectory: changing needs and recurrent engagement. Engagement in support-seeking depended on the alignment of readiness, recognition of need, and relational safety. This model offers a framework to improve how post-treatment support is designed and delivered in practice.

## Simple Summary

Young people who have had cancer often continue to experience emotional, social, and physical challenges long after treatment ends, yet they do not always seek or use the support available to them. This study worked with young people with lived experience of cancer to create a model which explains the processes involved in support-seeking. Support was more likely to be sought when young people recognised a need for help, felt emotionally ready to receive it, felt confident that support would be safe, and coming from a trusted person or organisation. We will use this model to guide improvements in survivorship support. The next step is to work with healthcare teams, charities, and young people to evaluate how these ideas can be applied in practice, and to see whether this will improve access to support.

## 1. Introduction

Adolescents and young adults (AYAs), typically defined as individuals aged 15 to 39 years at diagnosis [1], represent a distinct population within oncology. In the United Kingdom (UK), the term ‘teenage and young adult’ (TYA) is also commonly used in clinical service provision and by charities, typically referring to those aged 15–24 years, although UK research may still use the wider age range. Despite improvements in survival rates for this group over recent decades, with 5-year survival rates exceeding 80%, AYAs continue to experience complex and multifaceted challenges following cancer treatment [2,3,4]. Exposure to chemotherapy and radiation is associated with an increased risk of secondary malignancies, cardiovascular disease, and other long-term complications affecting pulmonary, neurological, and endocrine function [4]. These physical side effects can intensify psychological and developmental challenges in AYAs during critical stages of emotional, social, and cognitive growth. The resulting emotional distress, body image issues, and disruptions to social reintegration may further complicate this already transitional stage of life, negatively impacting self-esteem, interpersonal relationships, and adjustment to adulthood [5,6]. Evidence suggests that AYAs face a high rate of physical, psychosocial, and practical concerns and are often not seeking or receiving support to address these. This is reflected in wider AYA cancer research, including large-scale studies such as the BRIGHTLIGHT study [7] and European initiatives such as STRONG AYA [8], which highlight variation in care experiences and outcomes, and therefore the need for more consistent, age-appropriate support across systems. On average, 43% of AYAs reporting a concern sought support [9]. Common reasons for not seeking support included not wanting to ask, being told that it was normal to feel the way they did, or embarrassment. Among those who did seek support, 37% reported difficulties in accessing the support they needed [10].

A mixed methods study of young adults aged 19–26 years [11] reported that young people need to be motivated and self-aware to seek support and lack of initiative to do this was a barrier to receiving support at the end of treatment. For some young people in this study, asking for help did not come naturally to them, or did not match their personality style to discuss or seek support to deal with their emotions [11]. The study proposed that assessment and identification of psychosocial needs via holistic needs assessments should be routinely integrated into follow-up care; however, findings from this study suggest that this is not routinely implemented.

Two studies [12,13] exploring barriers to accessing psychosocial support in AYA cancer survivors identified several other key challenges which were more evident post treatment [12]. Person-centred barriers included lack of motivation, being too sick to engage, and practical issues, while service-related barriers involved a lack of appropriate, available and accessible support [12]. AYAs also reported that, while they might recommend support programs to others, they struggled to commit themselves, due to resistance to trying something new amid the demands of medical treatment [13]. Systemic factors included services not being offered, inconsistent service provision and poor integration between healthcare and community settings [12].

Facilitators to support access included AYAs having good coping skills, approaches which emphasised AYAs’ self-motivation and agency, and offering programmes to everyone and at multiple timepoints [13]. This is consistent with other research [14] which states that young people value flexible and repeated offerings of support over time, recognising that specific needs become more focal at different stages of life and that individuals follow different trajectories.

A significant challenge in AYA cancer care post treatment lies in the evolving and variable nature of their support needs. These needs often do not always emerge immediately after treatment but instead become more apparent at key life transitions, such as starting university or entering the workforce. Many AYAs initially prefer to manage their recovery independently, relying on informal support networks rather than formal services [14]. However, the reactive nature of current follow-up care, which often waits until AYAs are in crisis, highlights the gap in proactive, accessible support [14]. Furthermore, AYAs often describe a delay in their readiness to seek support, recognising that the need for support becomes more pressing only at specific life milestones. This delayed engagement suggests that AYAs may require time to adjust to life after treatment before they are willing to seek emotional or social support from professionals or peers [14].

Despite growing awareness of these barriers, a significant proportion of AYAs delay or avoid seeking support even when experiencing substantial distress or impairment. This challenges assumptions that improving service availability or support service awareness alone will sufficiently address unmet needs. Instead, it suggests a more complex interplay between individual readiness, social influences, and health system factors; that remains underexplored in the literature.

**Figure 1:**
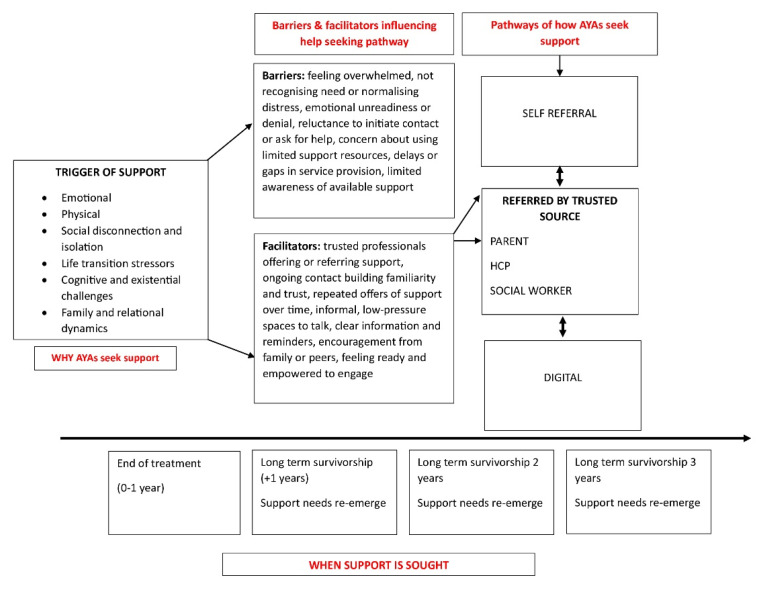
Preliminary model.

Drawing on findings from our previously published qualitative study exploring post-treatment support experiences among TYAs aged 16–25 years who had completed cancer treatment 1–6 years earlier [14], we developed a provisional conceptual model of support-seeking (Figure 1).

This present study aimed to refine and further develop this provisional conceptual model of support-seeking post treatment, in collaboration with young people with lived experience of cancer, as part of a patient and public involvement (PPI) approach. The objective was to ensure that the model reflects lived experience and supports the development of relevant, usable survivorship support.

## 2. Materials and Methods

### 2.1. Ethical Considerations

Ethical approval for this PPI activity was obtained from the University of Southampton ethics committee (ERGO 108296). Written informed consent was obtained prior to their involvement, and ethical guidelines were adhered to ensuring confidentiality, voluntary participation, and the right to withdraw at any time. The young people research partners received an information sheet and contact details for support if needed.

### 2.2. Design

An iterative qualitative co-production design was employed, using mixed methods (survey and workshop) to develop and refine a conceptual model. Reporting follows the Guidance for Reporting the Involvement of Patients and the Public short form checklist (GRIPP2) [15] (Supplementary File S1).

This work represents an adaptation of the Generative Co-Design Framework for Healthcare Innovation [16], with emphasis on the co-design and post-design phases to iteratively refine a conceptual model of support-seeking. The framework comprises three stages: Stage 1 (pre-design), involving contextual understanding and preparation; Stage 2 (co-design), involving collaborative exploration and generation of ideas; and Stage 3 (post-design), involving analysis and translation of findings into outputs.

Young people with lived experience of cancer were involved as PPI partners, providing experiential feedback, refining the structure and content of the model, and contributing to recommendations.

### 2.3. Stage 1: Pre-Design (Contextual Grounding and Preparation)

Stage 1 was informed by a prior co-design study that developed a preliminary conceptual model of support-seeking, outlining the types of support sought, pathways to accessing support and, barriers and facilitators influencing engagement with support [14] (Figure 1). Stage 1 comprised two phases: (1) semi structured interviews and co-design workshops with TYAs with lived experience of cancer, and (2) feedback from healthcare/allied health professionals (HCAPs) where we explored their perspectives and experiences of support post treatment, decision making around support provision, and their preferences for how this support should be delivered. This provided contextual grounding for the current refinement of the model.

#### PPI

We refined and expanded this model using data from young people diagnosed with cancer aged between 16 and 39 years. Expanding the age range ensured the model reflects the full AYA population and captures how differences in life stage, social roles, and responsibilities such as career, relationships, and family affect when, why, and how young people seek support after cancer. Young people were eligible if they had lived experience of cancer between the ages of 16–39, were able and willing to provide informed consent, and were fluent in English. No additional exclusion criteria were applied beyond these eligibility requirements. Co-design workshops involved members of the University of Southampton’s Young People with Lived Experience of Cancer Advisory Group (YOUNG CAN-LEAP), led by SCS and NC. All PPI participants were recruited through this established advisory group. PPI members were supported in their involvement to ensure safe participation through the workshop being conducted by an experienced member of the research team and opportunities to pause, take breaks, or withdraw at any time. At the end of each workshop, PPI contributors were provided with a debrief sheet containing signposting to support if needed, and details for follow-up contact should they have further questions.

### 2.4. Stage 2: Co-Design (Data Collection and Collaboration)

#### 2.4.1. Online Survey

**Figure 2:**
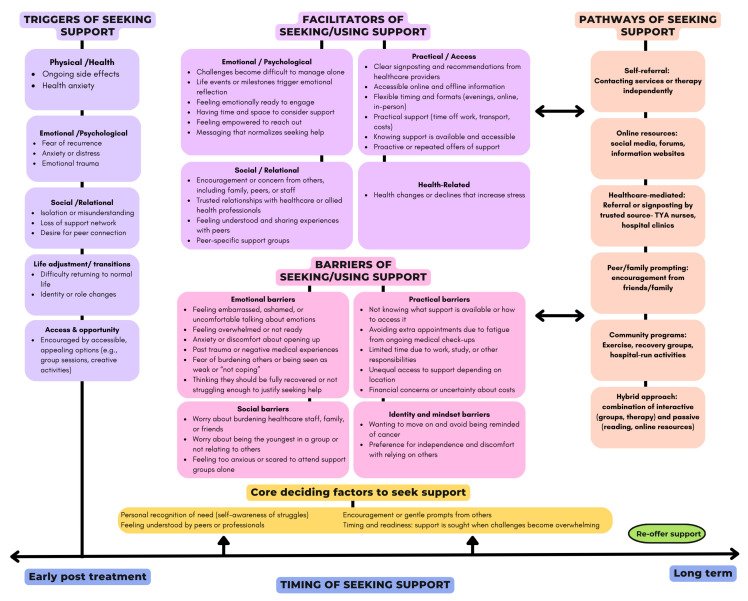
Model drafted from survey responses and Support My Way findings.

Socio-demographic characteristics (age at diagnosis and enrolment) were self-reported prior to workshops. An online survey, conducted via Qualtrics, included 12 open-ended questions to gather detailed feedback on young people’s experiences of seeking support after cancer treatment. Questions explored triggers for seeking support, reasons for not seeking support, potential factors that might encourage support-seeking in the future, preferred pathways for accessing support, timing of support needs, and the most important types of support. The survey also asked young people to reflect on the preliminary support-seeking model and suggest improvements to better reflect the realities of young people’s experiences. Survey responses were used to refine the model (Figure 2) prior to the co-design workshops.

#### 2.4.2. Co-Design Workshops

The workshops adopted a participatory, co-design approach [17], engaging members of the YOUNG CAN-LEAP advisory panel [18] and was conducted using Microsoft Teams. Young people completed the online workshop from their homes. NC, a female researcher with extensive experience of qualitative research on topics related to cancer led the workshop. The young people were previously known to the researcher through their role on the advisory panel. The workshop lasted 1 h, was video recorded and auto transcribed through Microsoft Teams. Field notes were written following the workshop.

Prior to the workshops, the young people were emailed the draft model (Figure 2) to allow time for reflection on the model so that they could feel prepared to discuss in the workshop. Workshops began with an overview of the aims, followed by a structured discussion of the model and related survey responses to explore experiences in greater depth. The young people then discussed recommendations for third-sector organisations and healthcare professionals (HCPs) to improve post-treatment support pathways. The workshops concluded with a discussion of potential dissemination strategies and final reflections.

### 2.5. Stage 3: Post-Design

#### Data Analysis

**Figure 3:**
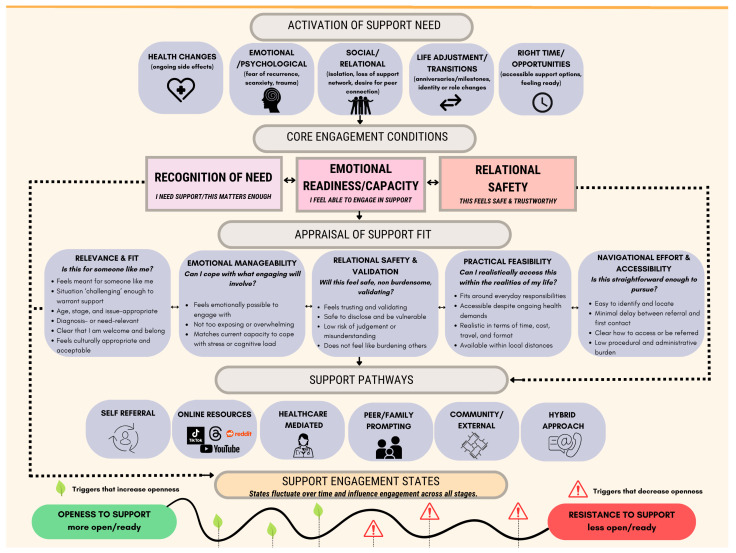
Model of support-seeking in young people with lived experience of cancer (final version). This model shows how young people engage with post-cancer treatment support as a dynamic, process influenced by readiness, context, and time. Support needs are activated by health changes, emotional or psychological distress, social factors, life transitions, or timely exposure to support. Engagement is shaped by three key conditions: recognising a need for support, feeling emotionally ready, and perceiving support as safe and validating. These conditions do not all need to be in place at initial contact; one or more may prompt awareness, enquiry, or tentative engagement. However, more sustained and meaningful engagement is most likely when all three are established. When one or more of these conditions are present, young people assess whether support fits their situation, considering relevance, emotional manageability, relational safety, practical feasibility, and ease of access. If it feels appropriate, they access support through multiple pathways such as self-referral, healthcare services, online resources, peers, or a combination of routes. Engagement changes over time, with individuals moving between periods of openness to support and resistance to support as their needs and readiness shift.

Survey responses and co-design workshop data were analysed by NC and SS using a deductive, descriptive thematic approach guided by Naeem et al.’s [19] systematic process for developing conceptual models in qualitative research. Workshop transcripts were first cleaned by NC to ensure the accuracy of the auto transcription and then read repeatedly to support familiarisation with the data and identification of key quotations relevant to the aims. Survey responses were summarised descriptively to identify patterns in triggers, barriers, facilitators, timing, and support pathways, which informed the second preliminary conceptual model (Figure 2), and guided subsequent analysis of the workshop data to create the final model (Figure 3).

Key words and phrases reflecting young peoples’ experiences and perspectives were identified across both datasets and used to inform coding. A deductive coding framework, based on the preliminary model and survey findings, was applied to the workshop transcripts while allowing additional codes to be identified where new perspectives were introduced. Coding was conducted systematically across the dataset. Related codes were then grouped to develop broader themes representing key aspects of support-seeking.

Themes were subsequently interpreted to identify underlying concepts and relationships describing how young people engage (or not) with post-treatment support. These concepts were compared and refined across both the survey and workshop data to ensure the model reflected patterns evident across the dataset. The resulting concepts were then integrated to refine the conceptual model. The draft model was then shared with the advisory panel as a co-design activity to check that the interpretation resonated with their perspectives and experiences. Subsequent feedback was sought by email after the co-design workshop to ensure interpretations were accurately conveyed. Panel feedback contributed to further refinement of the model, including clarification of key concepts, strengthening the emphasis on readiness, recognition of need and relational safety, and ensuring the model reflected the non-linear and evolving nature of support-seeking. Any differences in interpretation were discussed and resolved through consensus between authors (NC, SS, AK, NG, TI, DW). Excel was used to manage the data.

## 3. Results

A total of 12 members of the advisory panel were invited to participate in the survey, of which eight expressed interest and one subsequently decided not to participate. Seven patient partners in the advisory panel completed the online survey and four advisory panel members took part in the online co-design workshop. The age range of the young people from both phases at enrolment was 19–43 years and at diagnosis was 18–38 years.

Analysis of survey responses and co-design workshop discussions identified four conceptual themes that illustrate young people’s experiences of seeking support after cancer treatment. These themes map directly onto the model’s previously developed components—triggers, barriers, facilitators, pathways, core deciding factors, and timing—providing a nuanced understanding of support-seeking among AYAs with cancer (see Figure 3).

### 3.1. Readiness to Engage: Recognition, Emotional Readiness, and Relational Safety

#### 3.1.1. Model Stage: CORE ENGAGEMENT CONDITIONS

Young people described engagement with support as a staged process shaped by recognising a need, feeling emotionally ready, and believing support would be safe and understanding, although these factors did not always need to be aligned at the point of first contact. Initial engagement could be prompted by one or two conditions, such as recognising a need, encouragement from a trusted person, or access to a familiar service. Emotional readiness and trust in support could develop over time, shaping whether early contact progressed into sustained and meaningful engagement; where these were not yet in place, engagement was often delayed, tentative, or limited.

Recognition of need often developed gradually; “…I didn’t want my normal life to be disrupted again by cancer because I had finished treatment… it takes a while to admit how big that life experience was” (HS01). Some young people noticed it themselves only after difficulties became hard to manage, while for others, family or close friends identified changes first: “For me I got support when things started getting too much and my mum was noticing” (HS06). Support became more likely when young people reached a threshold where the challenges could no longer be minimised or managed privately. Recognition could be relationally mediated, with trusted others helping to identify when support might be needed, particularly where young people themselves were still trying to contain or normalise their distress. However, while others could notice the need for support or signpost young people to services, this did not automatically lead to support seeking. Young people emphasised that they needed to personally recognise the need for support and feed ready to act, describing support-seeking as requiring a degree of internal motivation or agency., One young person reflected, “I think ultimately personal recognition of need is the most important… I was told for ages by my parents to try a support group but due to time constraints, nerves, I didn’t go to one for a long time. I had to be the one to find the internal motivation to get myself there and to engage with my peers at the group… It didn’t matter how much people were telling me, I can see you’re struggling. It’s until you’re ready…” (HSW01).

A central feature of this theme was the importance of emotional readiness. Young people described a gap between knowing support might help and feeling able to access it, especially when cognitive and emotional load was high: “…I think… you can have all the information given to you, because when you’re going through treatment, you are bombarded with information the whole time and it does take a while…to get to the point when you are then ready to actually pick up that paperwork again and go, okay, what was actually available for me? I feel ready to do that” (HSW01). Support had to align with a point at which it felt emotionally and cognitively manageable to receive. Emotional readiness could be activated or intensified by stress points such as scan appointments, cancer-related anniversaries, changes in health, or key life milestones (e.g., starting university or a new job): “Even now, I tend to try to power through challenges on my own and bottle things up… I might consider [seeking support] if I reach a point where the emotional or physical impact becomes harder to manage alone, like significant changes in my health, prolonged emotional struggles or a milestone that brings up unexpected stress or anxiety” (HS02).

Relational safety and validation strengthened engagement. Support felt meaningful when it resonated with lived experience and came from someone trustworthy and emotionally attuned: “The biggest thing that makes a young person reach out for support is feeling like someone truly understands what they’re going through” (HS02). Recommendations from trusted relationships were particularly influential: “I’m really close to my parents as well. And it was kind of coming from them, I think was not more valuable, but I would take more notice of it because they know me so well…I think it would come better from somebody who knew me really like inside and out, basically” (HSW03).

#### 3.1.2. Model Stage: ACTIVATION OF SUPPORT NEED

Challenges across multiple domains acted as triggers that highlighted the need for support. Physical and health-related concerns included ongoing side effects and health anxiety, while emotional and psychological factors were especially prominent, such as fear of recurrence, anxiety, and emotional trauma. Social and relational difficulties also prompted support-seeking, including feelings of isolation, loss of support networks, or a desire for peer connection. Life adjustments and transitions, including returning to normal routines and shifts in identity or role after diagnosis and treatment, created further pressure. Access influenced engagement too, with young people more inclined to seek support when options were appealing or accessible. These triggers made the need for support more salient, and engagement was more likely when recognition of need, emotional readiness, and relational validation were present, individually or in combination, making support feel personally acceptable and actionable.

### 3.2. Access and Appraisal of Support: Visibility, Fit, Feasibility, and Burden

#### Model Stage: APPRAISAL OF SUPPORT FIT

Accessing support was shaped by a range of informational, practical, emotional and procedural barriers that interrupted or prevented engagement, even when young people recognised a need. Young people described multiple barriers that limited both the pursuit of support and the ability to sustain engagement once support options had been identified. These barriers operated across overlapping domains, suggesting that access was shaped by whether services existed, their visibility, practical accessibility, emotional manageability, and perceived relevance to the young person’s circumstances.

A key barrier was limited knowledge of what support existed, how to access it and whether it was appropriate for young people in the post-treatment phase. Young people described difficulty locating relevant services, uncertainty about eligibility, and concern that available support would not feel age-appropriate or relatable. Unequal access depending on location further intensified this, noting that what was available varied substantially by hospital, region, or local area, “It’s really hard to find stuff.” (HS04). Uncertainty about whether a service was intended for someone at their stage post treatment also created hesitation and could discourage engagement before contact was even made: “And another thing for me about [cancer charity] was I wasn’t entirely clear because I was post treatment and kind of transitioning back to normal. I wasn’t entirely clear whether [charity] was for people that were going through cancer. So I didn’t want to kind of be rejected when I got there” (HSW03).

Practical and structural barriers further restricted access. Some young people described support as difficult to fit around work, daily responsibilities, and ongoing efforts to return to normality after treatment. This was particularly evident where services were only available during standard working hours: “All the services… are all during the day… I work four days a week… so I can never access any of the support groups” (HSW03). For some, fatigue from ongoing medical appointments also made the idea of adding further support sessions feel burdensome, particularly when they were already exhausted by regular follow-up care. Financial concerns or uncertainty about whether support would involve costs could create further hesitation, even when young people were otherwise interested.

Emotional and psychological barriers also influenced engagement. Young people described feeling overwhelmed, not ready, or uncomfortable with the idea of opening up, particularly where support involved discussing emotions directly. Some young people questioned whether their needs were severe enough to justify support, or whether engaging would be worth the effort: “Not knowing whether support will be helpful or a waste of time, or whether you are struggling enough to warrant support” (HS07). Past trauma and negative medical experiences also influenced seeking support. For some, support linked to hospital settings or cancer-related conversations risked reactivating distress, particularly where prior treatment experiences had been traumatic: “Trauma from past medical experiences… wanting to escape from the healthcare environment and difficult experiences” (HS03).

Social and relational barriers further shaped whether support felt acceptable. Some young people worried about burdening healthcare staff, family, or friends, particularly when concerns felt uncertain, minor, or difficult to explain. Some deliberately avoided raising issues for fear of making others worry or of taking up too much professional time. Group-based support could also feel intimidating, especially where young people worried they would be the youngest person there, would not relate to others, or would have to attend alone when already felt anxious or vulnerable. Concerns related to whether the support space would feel emotionally safe and socially manageable. Perceived fit was relational as well as informational, with concerns about age, stage, confidence, and interpersonal burden all shaping willingness to engage.

Identity and mindset also operated as important barriers. Several young people described a strong preference for independence and discomfort with relying on others, even when they recognised a need for support. Some framed support-seeking as something they should avoid unless absolutely necessary, preferring to ‘power through’ difficulties on their own. Others expressed a desire to move on from cancer and avoid support that might force continued identification with illness or bring unwanted reminders of treatment. For these young people, seeking support could feel at odds with efforts to reclaim normality, autonomy, or a post-cancer identity.

Young people also described procedural and bureaucratic barriers that made access feel exhausting and, at times, unsustainable. Referral pathways, administrative requirements, long waits, and repeated retelling of personal histories could become barriers in themselves, particularly when young people were already emotionally depleted: “It’s like there’s some support to access. It’s like a real pain. Like you have to go through a few layers or get a referral or supply information or whatever. And actually, there’s that I just haven’t bothered because it just feels like it’s too much effort. Whereas actually, if my nurse could do some of it on my behalf. That would be helpful” (HSW02). Another barrier was delay in access, where time between requesting support, referral, and first contact reduced motivation or meant needs had changed by the time support became available. A related issue was limited information at the point of requesting help, where individuals could have been referred without being told what support options were available or what they involved, meaning they were referred without knowing what support involved, and only received this information weeks later, by which point the support was often less relevant or no longer felt necessary.

Finally, cultural and interpersonal factors could also influence whether support felt acceptable. Support-seeking could be framed within cultural or religious contexts as a sign of weakness, creating an additional barrier. As one young person explained, “I know having spoken to other people who come from different religious backgrounds that it can be seen as a sign of weakness if you try to access help so that that could be a barrier for people” (HSW01).

Young people described a mismatch between available support and the realities of post-treatment young adulthood, where services could be hard to find, hard to navigate, poorly timed, emotionally demanding, socially uncomfortable, or uncertain in relevance. Within the conceptual model, these barriers appear to interrupt movement from recognition of need to engagement, often redirecting young people toward self-navigation, informal alternatives, or disengagement altogether.

### 3.3. Pathways to Support: Multi-Modal, Layered, and Non-Linear Engagement

#### Model Stage: SUPPORT PATHWAYS

Young people navigated and assembled support through multiple, overlapping pathways rather than through a single linear route. Young people described support-seeking as a multi-modal and non-linear process, moving across formal, informal, and digital pathways in ways that were often gradual, selective, and shaped by emotional readiness, perceived relevance, and situational need. Young people often combined self-referral (such as independently contacting services or therapy), healthcare-mediated routes (including referral or signposting from trusted professionals such as TYA nurses or hospital clinics), peer and family prompted pathways (where encouragement from friends or relatives initiated support-seeking), community-based opportunities (such as exercise programmes, recovery groups, or hospital-run activities), and digital resources, including social media, online forums, newsletters, and information websites. Many young people also described a hybrid approach, drawing on both more interactive forms of support, such as groups or therapy, and more passive forms, such as reading online resources or observing social media content before deciding whether to engage more actively. Different pathways served different emotional, practical, and relational functions. As one young person explained, “Personally I would probably turn to friends first… If that felt too hard I would likely search online hoping to find advice or stories from others who have faced similar challenges” (HS02).

Young people described moving from passive exposure to more active participation, with repeated visibility and timely re-offering of support increasing the likelihood of uptake: “I think for me, the only person I’ve ever like, proactively kind of asked for support is my nurse or my oncologist. I think everything else for me has been like I’ve happened to see it at the right time. Yeah, so that thing about reoffering support regularly, it’s definitely been very true for me…I happened to see a kind of Facebook thing about like a Macmillan ad for kind of counselling sessions… But I like, yeah, it’s been kind of tried stuff like dipped in and out, like, yeah, like I did a group of art therapy. I saw that on the Shine newsletter” (HSW02). Another young person emphasised the importance of variety, noting that “All pathways are important and it is important to have a variety of them, some which involve engagement with people, and some which are more passive (e.g., online resources that can just be read and absorbed)” (HS07). Access of support was influenced through repeated exposure, and opportunistic encounters.

Pathway choice was also shaped by emotional safety and the desire to manage relational burden. Some young people deliberately selected routes that allowed them to seek reassurance or information without worrying family members or feeling that they were overusing professional support: “The closest family member I have in my life is my mum, but I wouldn’t necessarily go to her first if I started struggling because I wouldn’t want her to worry…so I probably try and seek some other route, like go to my CNS or my survivorship nurse first, and then once that’s kind of, at least in the process of being secured, then I would go to someone like my mum or like my friends and my family and just say like, this is what I’m doing” (HSW04). Similarly, another young person described turning to diagnosis-specific online communities rather than family or clinicians for questions that felt too minor, uncertain, or difficult to raise: “I’ve utilised the online forums…And depending on what the question is, would depend on which platform I will go to initially to ask for if anyone’s got any hints or tips or advice because I don’t like to burden my friends or my family or make them worry about things… it’s helped me network with other people that have been in my shoes because it’s great dealing with health professionals, but they are exactly that. They’re health professionals. They haven’t got the lived experience. When you talk to someone who’s been in your shoes, they can relate on a totally different level” (HSW01).

Digital and peer pathways were particularly important but were also described as emotionally ambivalent. Online forums, social media groups, and newsletters created opportunities for easy access, connection, and diagnosis-specific peer advice, especially when formal support felt inaccessible or overly burdensome. However, these same spaces could also become distressing, particularly when young people encountered more severe experiences at times when they were already feeling vulnerable. As one young person reflected, “I actually found it quite difficult to read some of the things that were going on in the Facebook group. Sometimes you come to it because you’re feeling a bit down or like you want support and then you read somebody’s going through something that’s really awful.” (HSW03). Another added, “And then you don’t want to engage with the pages as much if you’re kind of feeling a bit vulnerable yourself, but then you might miss other posts. It’s a hard balance I think” (HSW04). Digital support was a flexible option, but people had to judge when and how it felt safe to engage emotionally.

At the same time, young people described significant variability in how easy these pathways were to identify and navigate. Online directories were often experienced as inconsistent or outdated, particularly when trying to locate age-appropriate or diagnosis-relevant support: “I think there are so many online places where you can put in your postcode and see what the support services are, but so many of them are so out of date…. I couldn’t find any support groups for anyone of my similar age and it was quite challenging” (HSW01). Awareness among HCPs of specialised programmes could also be limited, further reducing visibility of relevant options: “My nurses didn’t even know about the Battle Cancer programme… I told them about it because they didn’t know anything. (HSW02).” While pathways were multiple, they were not always clearly signposted, meaning that young people often had to piece together support routes themselves.

Support-seeking involved trying, adjusting, and switching between options. Young people moved between different pathways depending on what felt manageable, accessible, and relevant in the moment. Access was facilitated when support was clearly signposted, repeatedly offered, endorsed by trusted professionals, available in flexible formats, and delivered through pathways that felt emotionally safe and personally relevant.

### 3.4. Support Trajectory: Changing Needs and Recurrent Engagement

#### Model Stage: SUPPORT ENGAGEMENT STATES

Support needs shifted over time and often recurred at different points in survivorship, showcasing the temporal nature of engagement. Young people described their needs as evolving; for some, the effects of treatment were experienced as ongoing, with the type and intensity of support needed shifting over time. Early after treatment, one young person emphasised physical recovery, fatigue, and the immediate shock of what had happened, while later needs were more likely to relate to emotional adjustment, social reintegration, return to work or education, and uncertainty about the future: “I think the need for support changes over time after treatment because the effects of cancer don’t just stop once treatment ends they can last a lifetime At first it might be about coping with the physical side effects and shock of what’s happened. Later on it can be more about managing long term challenges returning to work or school or dealing with ongoing worries” (HS02).

Openness to support was episodic and often triggered by specific events, changes, or milestones. Young people highlighted how distress could intensify around anniversaries, scans, changes in health, or key life transitions, creating periods where support became more salient or urgent even months or years after treatment. Support need was not consistently present at the same intensity, but emerged in response to changing circumstances, reinforcing the importance of understanding support-seeking as recurrent and situational rather than one-off.

A prominent timing issue was the mismatch between when information or support was offered and when young people felt able to process or use it. Resources offered too early could be effectively inaccessible, even when technically provided. Timing and repeated exposure also shaped whether support became actionable. A few young people described needing to encounter support opportunities more than once before feeling ready to engage: “Seeing something and being in the right frame of mind to access it. It took me 3 times of seeing a FB ad for counselling through Macmillan, each time filling out a bit more of the form, or ignoring the callback and then finally answering to actually access it. (HS04)”. Engagement could be incremental rather than immediate, and gentle, repeated, and non-pressured offers of support were often more effective than one-off or overly forceful encouragement. This was reinforced by another young person’s reflection that support could not be imposed externally: “…I think it has to come from us. But if you talk it through with us, then maybe you can help us come to our own conclusions. I don’t think it’s something that can be forced necessarily. It has to come organically. But by just talking through things, […] coming to our own conclusions…” (HSW03).

Openness to support increased and decreased over time, influenced by specific stress points, and often became actionable only when support was offered in a way that aligned with readiness and context. The conceptual model positions time as ‘openness to support’ and ‘resistance to support’, an active part of the model shaping when support is recognised, when it is usable, and when re-engagement becomes possible.

## 4. Discussion

To our knowledge, this is the first study to develop a co-designed conceptual model of support-seeking after cancer treatment among young people with lived experience of cancer. The findings suggest that support becomes usable under one or more particular conditions, including when difficulties are recognised and emotionally acknowledged, when seeking support feels legitimate and safe, when available options are perceived as relevant, and when pathways to access are visible and easy to navigate. These conditions position post-treatment support-seeking as a dynamic process in which young people move in and out of readiness over time and assemble support across formal, informal, peer, and digital routes.

A central finding across the workshop themes is the importance of readiness as a condition for engagement. Support may be visible, available, and encouraged by others, yet remain unused until individuals reach a point of recognition and emotional willingness to act. This aligns with Nuzulullail et al.’s [20] scoping review who found that cancer survivors’ transition readiness varies across diagnosis, treatment, and survivorship phases, and is shaped by interacting individual and structural factors including age, insurance status, emotional and physical state, treatment status, cancer stage, patient competence, access to health services, and the quality of information and communication. Survivorship was identified as a particularly important adjustment period, characterised by emerging support needs and fluctuating levels of preparedness for transition. Baker et al. [21] provide a useful comparison, finding that willingness to engage with emotional support varied across the cancer trajectory, with patients recently diagnosed often reluctant to acknowledge or address emotional needs, despite indications of distress, whereas those further along in treatment were more open to discussing and receiving support. While their study focused on older adults with cancer and the present work focuses on young people after treatment, both highlight how engagement with support varies across the cancer trajectory, with openness to emotional support shifting over time in relation to changing experiences of distress and adjustment.

These patterns can be further understood through Self Determination Theory (SDT) [22,23,24] which explains motivation, psychological growth and wellbeing in terms of satisfaction of three psychological needs: autonomy, competence, and relatedness. From this perspective, readiness may reflect a point at which young people experience support-seeking as self-endorsed (autonomy), feel able to understand and manage their situation (competence), and perceive support as emotionally safe and non-judgmental (relatedness). Where these conditions are not met, support may not be accessed despite being available. Thus, ‘readiness’ can be understood as a timing- and needs-based process influencing engagement with support.

Young people assemble support across formal, informal, peer, and digital pathways, engaging with support when it feels safe, manageable, and relevant. Support-seeking appeared to involve moving between and combining different formats depending on the nature of need, emotional state, and perceived suitability. Drawing on a previous linked study (SUPPORT MY WAY) [14] that interviewed young people about their preferences when they needed support after treatment, and in what format; young people accessed support from a range of sources, including HCPs, peer networks, and charities, with individuals selectively engaging with different formats based on perceptions of emotional safety, accessibility and legitimacy. This pattern reflects the way psychological needs are negotiated across different contexts. Autonomy is expressed through active selection of support sources with young people deciding when to seek formal input, rely on peers or use digital or informal spaces. Competence is evident in the ability to navigate and integrate the variety of types of support, particularly when individuals learn which formats are most effective for different types of needs, such as practical advice, emotional processing, or reassurance. Relatedness is closely tied to perceptions of relational safety, with engagement shaped by whether interactions feel approachable, non-intimidating, and emotionally attuned, whether from professionals, peers, or informal networks. Variability in format use therefore reflects an ongoing process of matching psychological needs to the type of support that best fits them at the time.

Time also emerged as a key determinant of engagement, shaping when support becomes accessed. Across accounts in this work, engagement with support was dependent on alignment between the timing of support provision and patients’ evolving needs throughout the survivorship trajectory. A qualitative study that interviewed 36 young women breast cancer survivors under the age of 50 (a third of whom were experiencing social deprivation) [25] similarly found that one-off signposting and static information were often insufficient, as they did not accommodate changing post-treatment needs, particularly in relation to body image, psychological wellbeing, and professional rehabilitation issues. Patients require repeated opportunities to revisit and re-engage with support as new concerns emerge over time. Through an SDT lens, as individuals move through different phases of survivorship, the salience of the core needs for autonomy, competence and relatedness are likely to shift in response to newly arising physical, emotional and social challenges/demands. Therefore, one-off or poorly timed information provision may be insufficient to support autonomy, leaving patients unable to make informed decisions when needed. It may also constrain competence by limiting the knowledge or confidence required to navigate new challenges, and reduce relatedness, particularly when opportunities for ongoing emotional support are restricted. In contrast, models of care that incorporate flexible, iterative and accessible forms of support are better positioned to respond to these shifting psychological needs, and therefore facilitate more sustained engagement with survivorship services [14].

### 4.1. PPI Involvement

Patient and public involvement through co-design was central to the development of the conceptual model and influenced both its structure and interpretation. The involvement of young people with lived experience helped ensure that the model reflected how support-seeking is understood and experienced in practice, particularly in relation to readiness, relational safety, and the non-linear and evolving nature of engagement with support. PPI input helped to refine the language and framing of key concepts, ensuring they aligned with how young people describe their experiences. A further strength of this approach was that iterative feedback enabled alignment between the preliminary model and young peoples’ lived experiences, supporting a more nuanced representation of survivorship support-seeking. A key challenge of this approach was balancing all perspectives within group-based discussions. To address this, follow-up feedback via email between stages provided an additional route for young people to share more considered and nuanced reflections to shape the model.

### 4.2. Strengths and Limitations

Several strengths are identified, including a novel, co-designed conceptual model of post-treatment support-seeking among young people with cancer, grounded in lived experience, and providing a dynamic account of how engagement evolves over time. However, some limitations should be considered. The number of young people partners was relatively small and lacked diversity, which may limit the transferability of the findings. PPI partners were self-selecting and drawn from an established advisory group, and may have been more attuned to recognising need and engaging with support, potentially underrepresenting those who are less aware of, or less willing to seek support. While the co-design approach strengthened the relevance and credibility of the model, the group-based nature of data collection may have shaped how experiences were articulated and prioritised, for example through shared reflection and discussion dynamics. Future research should seek to include more diverse and less engaged populations to further test and refine the model.

### 4.3. Implications for Practice

This work offers a framework for understanding the complex nature of support-seeking and support needs during a challenging life stage, and translates this into actionable recommendations for practice, written with and reviewed by our PPI partners (see Table 1). These findings suggest that improving post-treatment support for young people may depend more on ensuring that existing support is experienced as visible, relevant, and accessible at the right times. Using an SDT-informed perspective, this involves designing services that actively support autonomy, competence, and relatedness. HCAPs should move beyond one-off signposting and instead re-offer support repeatedly across survivorship. Signposting should explicitly clarify age range, stage of diagnosis, relevance, eligibility, format, and what initial engagement involves, as ambiguity around ‘fit’ may undermine perceived competence and deter access before contact is made. Referral pathways should be simplified where possible through clear handovers, supported self-referral, and reduced administrative burden. This should be supported by ensuring clinicians are actively aware of available charities and support services, with up-to-date information systems in place to enable consistent and informed signposting to patients.

Artificial Intelligence and digital technologies could support HCAPs and charities in keeping information up to date through centrally managed digital systems, ensuring signposting to relevant services is accurate and consistent across websites, clinical data, and follow-up information [26,27]. It could provide personalised signposting and summaries [26,28] using information already collected from young people (for example through routine assessments or questionnaires) to match needs to appropriate support, with clear links or contact details. It could also be used to re-offer support over time through automated but personalised reminders or conversational interfaces (e.g., check-ins or prompts based on time since treatment), with the option to revisit information or access human support if needed, while preserving choice and autonomy for the young person [29].

Charities and other support organisations should ensure that information is current, specific, and easy to navigate, while offering low-threshold, flexible formats that allow gradual engagement, including passive (where support can be accessed without actively initiating contact) and non-disclosing (without needing to share personal information or identify oneself) entry points. Such approaches may support autonomy by allowing individuals to engage on their own terms, while also enhancing competence through clarity and accessibility. Family members and trusted others may also play an important role by gently re-offering support over time and assisting with navigation in ways that support, rather than undermine, autonomy and relatedness. Future work should test these recommendations in practice and assess whether SDT-informed service changes improve engagement, accessibility, and perceived usefulness of support. Future work could also focus on developing and evaluating interventions that apply these principles within healthcare and third-sector pathways.

## 5. Conclusions

Post-treatment support-seeking in young people with lived experience of cancer is a dynamic process of recognising need, evaluating support, and selecting pathways that feel emotionally safe, personally relevant, and practically manageable in everyday life. Engagement is shaped by the interaction of internal readiness, emotional capacity, relational validation, perceived fit, access conditions, and changing circumstances over time. Young people use multiple, layered pathways, including formal, informal, peer, and digital routes, drawing on different forms of support for emotional, practical, and relational needs. Support needs also evolve and recur across survivorship, becoming more salient at specific stress points, transitions, and milestones. Findings highlight the need for flexible, responsive support that is re-offered at key stages and aligns with young people’s changing readiness, contexts, and points of need.

## Figures and Tables

**Table 1:** Recommendations for HCPs/AHPs, third-sector organisations.

Recommendations	What You Can Do
**Make support visible, approachable and relatable across settings (Competence & Autonomy)** Provide clear, step-by-step information on what support exists, who it is for/not for (making exclusions clear), and how to access it. Place information across clinic rooms, waiting areas, discharge summaries, follow-up letters, websites, and social media. Use simple prompts in clinical spaces (e.g., cards, posters, QR codes in treatment and waiting areas). Offer both self-referral and clinician-referral routes.	**Make support visible in the places young people already are in** Put short, friendly ‘support exists’ cards in treatment rooms, blood-test areas, and waiting spaces. Example: business-card sized prompts saying “Feeling off after treatment? Here’s where to start.” with a QR code. Add support links into appointment text reminders and follow-up messages. Include a “Support After Treatment” page in every discharge summary with clear steps and named contacts. Create a single, easy-to-navigate online hub for all post-treatment support. Embed a regularly updated online resource hub of relevant charities and support organisations within clinical systems or intranet pages, maintained by a designated service or coordinator, to support consistent signposting from healthcare/allied health professionals.
**Build repeated and proactive contact across the care pathway (Autonomy, Competence & Relatedness)** Offer planned emotional check-ins after treatment (e.g., 3 months and 12 months). Re-offer support over time as needs and readiness change. Use gentle follow-up messages to keep support visible without requiring initiation. Embed signposting into routine clinical interactions.	**Offer support at different time points, not just right after treatment** Schedule a 3-month and 12-month emotional check-in call as standard, not just medical appointments. Send a gentle, supportive message two months after treatment: “If life feels harder now than during treatment, that’s common. Here are some options if you ever want them.” Run quarterly ‘Life After Treatment’ workshops for those who were not ready earlier. Repeat signposting across multiple appointments because readiness changes over time.
**Strengthen continuity through trusted relationships (Relatedness)** Assign a named contact at the end of treatment. Maintain continuity of contact for at least 12 months post-treatment. Use existing clinical relationships to introduce post-treatment support. Support the transition rather than treating it as an endpoint.	**Maintain continuity and trusted support across transition** Give each young person a named key contact for at least one year post-treatment. Provide a physical or digital ‘After Treatment Toolkit’ including: ο Fear of recurrence information; ο Local and online support options (e.g., group, one-to-one, mentoring); ο Peer stories from other AYAs; ο Practical guidance (work- rights under the Equality Act, what employers can and cannot do, exercise, fertility, intimacy, finances) Build a simple “If you’re feeling X, here are your options” guide for staff to use in clinics. Train all staff (not just specialists) to give brief, supportive signposting conversations.
**Offer flexible, low- to high-intensity routes into support (Autonomy, Competence and Relatedness)** Provide a range of formats (group, individual, peer, digital, self-guided). Include low-pressure, optional-talking environments. Offer gradual entry routes into more formal support. Provide anonymous or self-directed options where needed.	**Create flexible pathways from informal to formal support** Run sessions where talking is optional (e.g., pottery, photography walks, creative groups), and clearly communicate this from the outset so individuals can choose how and when to engage. Create a ‘slow open’ counselling route starting with messaging, then phone, then face-to-face if chosen. Develop a menu of support options (groups, counselling, podcasts, videos, workbooks) Create anonymous ‘ask a psychologist’ text or web-based submission boxes. Produce short social media content (e.g., TikTok/Instagram series) showing what support actually looks like. Run ‘Mind + Body Recovery Days’ combining movement with short peer interaction.
**Develop age-appropriate, identity-safe peer support (Relatedness)** Prioritise AYA-specific spaces. Create informal, interest-based activities not centred solely on cancer. Use peer facilitators to bridge staff and young people. Reduce anxiety about joining for the first time.	**Build age-specific peer options that do not feel clinical** Create relaxed meet-ups such as art nights, hiking groups, board games, film clubs, and yoga or movement sessions. Use trained young facilitators or peer mentors. Offer buddy meet-ups or supported first attendance for those nervous about coming alone. Provide short peer-led introductions explaining “what groups are actually like”. Offer opportunities to meet the organisers in advance, before attending the group for the first time.
**Reduce practical and structural barriers (Autonomy & Competence)** Offer flexible timing and hybrid delivery. Reduce financial and logistical barriers. Ensure clear information about access and eligibility. Maintain safe digital spaces.	**Make access to support easier and more flexible** Offer evening and weekend groups where possible. Provide hybrid (online and in-person) attendance options. Fund or reimburse travel costs where needed. Ensure eligibility criteria are clearly explained in plain language across all materials.
**Support variability and long-term re-engagement (Autonomy, Relatedness and Competence)** Keep access open beyond immediate post-treatment phase. Allow people to pause and re-engage without barriers. Recognise that needs change across time and life stages. Provide flexible tools to identify current needs.	**Support the transition out of treatment intentionally** Keep services open so young people can return months or years later. Offer ‘any stage welcome’ groups that do not assume where someone is in their journey. Build an online tool that helps users identify what they need (emotional, social, physical, practical). Ensure pathways allow re-entry without re-referral or delay.

## Data Availability

The datasets used and/or analysed for this manuscript are available from the corresponding author on reasonable request.

## Supplementary Materials

The following supporting information can be downloaded at https://www.mdpi.com/article/10.3390/curroncol33070422/s1. File S1: GRIPP2 Checklist.
