# Supplementary material for: Mapping Support-Seeking After Cancer Treatment: A Co-Designed Model of Triggers, Timing and Support Pathways in Young People with Lived Experience of Cancer
Source: Curr Oncol. 2026 Jul 15;33(7):422. doi: 10.3390/curroncol33070422 (PMC13409497; doi:10.3390/curroncol33070422)
Supplement: Supplementary file 1 [file curroncol-33-00422-s001.zip › File S1 GRIPP2 Checklist.pdf]

Section and topic	Item	Reported on page No
1: Aim	Report the aim of PPI in the study	Page 3
2: Methods	Provide a clear description of the methods used for PPI in the study	Page 5
3: Study results	Outcomes—Report the results of PPI in the study, including both positive and negative outcomes	Page 16
4: Discussion and conclusions	Outcomes—Comment on the extent to which PPI influenced the study overall. Describe positive and negative effects	Page 17
5: Reflections/critical perspective	Comment critically on the study, reflecting on the things that went well and those that did not, so others can learn from this experience	Page 16/17
